# Motif-Aware PRALINE: Improving the alignment of motif regions

**DOI:** 10.1371/journal.pcbi.1006547

**Published:** 2018-11-01

**Authors:** Maurits Dijkstra, Punto Bawono, Sanne Abeln, K. Anton Feenstra, Wan Fokkink, Jaap Heringa

**Affiliations:** Department of Computer Science, Vrije Universiteit Amsterdam, Amsterdam, The Netherlands; Ottawa University, CANADA

## Abstract

Protein or DNA motifs are sequence regions which possess biological importance. These regions are often highly conserved among homologous sequences. The generation of multiple sequence alignments (MSAs) with a correct alignment of the conserved sequence motifs is still difficult to achieve, due to the fact that the contribution of these typically short fragments is overshadowed by the rest of the sequence. Here we extended the PRALINE multiple sequence alignment program with a novel motif-aware MSA algorithm in order to address this shortcoming. This method can incorporate explicit information about the presence of externally provided sequence motifs, which is then used in the dynamic programming step by boosting the amino acid substitution matrix towards the motif. The strength of the boost is controlled by a parameter, *α*. Using a benchmark set of alignments we confirm that a good compromise can be found that improves the matching of motif regions while not significantly reducing the overall alignment quality. By estimating *α* on an unrelated set of reference alignments we find there is indeed a strong conservation signal for motifs. A number of typical but difficult MSA use cases are explored to exemplify the problems in correctly aligning functional sequence motifs and how the motif-aware alignment method can be employed to alleviate these problems.

## Author summary

The most important functional parts of proteins are often small—but very specific—sequence motifs. Moreover, these motifs tend to be strongly conserved during evolution due to their functional role. Nevertheless, when trying to align protein sequences of the same family, it is often very difficult to align such motifs using standard multiple sequence alignment methods. Aligning functional residues correctly is essential to detect motif conservation, which can be used to filter out spuriously occurring motifs. Additionally, many downstream analyses, such as phylogenetics, are strongly reliant on alignment quality. We have developed a sequence alignment program named Motif-Aware PRALINE (MA-PRALINE) that incorporates information about motifs explicitly. Motifs are provided to MA-PRALINE in the PROSITE pattern syntax; it then scans the input sequences for instances of the pattern and provides a score bonus to matching sequence positions. Our method provides a reproducible alternative to editing alignments by hand in order to account for motif conservation, which is a tedious and error-prone process. We will show that MA-PRALINE allows the alignment of motif-rich regions to be fine-tuned while not degrading the rest of the alignment. MA-PRALINE is available on GitHub as open source software; this allows it to be easily tailored to similar problems. We apply MA-PRALINE on the HIV-1 envelope glycoprotein (gp120) to get an improved alignment of the N-terminal glycosylation motifs. The presence of these motifs is essential for the virus in evading the immune response of the host.

This is a *PLOS Computational Biology* Methods paper.

## Introduction

Sequence motifs are commonly described as relatively short conserved regions within a protein or DNA sequence [1]. These regions are of functional importance: they serve as binding sites for ligands or transcription factors, and as catalytic sites or structural elements. The presence of sequence motifs represents an additional conservation signal [2], in addition to the conservation of the amino acid sequence.

Accounting for motif conservation during sequence analysis is difficult. A conventional multiple sequence alignment (MSA) program will weigh each sequence position equally, scoring matches according to a substitution matrix such as BLOSUM [3] or PAM [4]. In a typical protein sequence, only a small fraction of amino acids are associated with a motif, which results in an underrepresentation of the conservation signal encoded by the motif. Cases even exist where traditional amino acid conservation is almost non-existent, such as with hypervariable regions. In these instances only the presence or absence of motifs is conserved.

**Fig 1:**
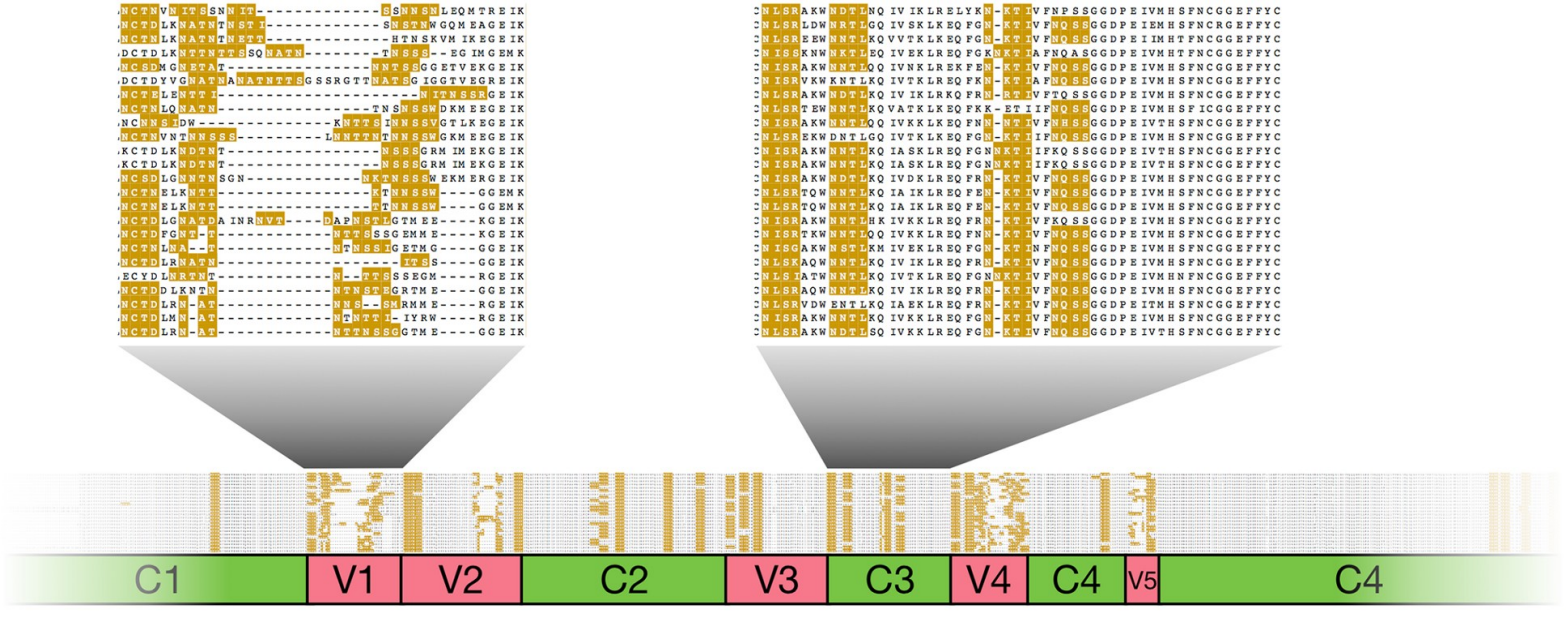
The problem of aligning motifs in variable regions: The HIV envelope protein (ENV) sequence [5]. The bottom strip shows an overview of the first half of the alignment. At the top, one of the variable (V1, left) and constant (C3, right) regions are shown. N-linked glycosylation is crucial for the immune evasion function of ENV; hence there is a prevalence of the *N*-{*P*}-*[ST]*-{*P*} motifs throughout. In C3 (right), the motifs are clearly well-aligned; however in the variable V1 (left), even though conserved flanking regions give a convenient anchoring for the alignment, it is obvious that the motifs are poorly aligned. This alignment was created with Clustal Omega [6]. For clarity, only a subset of representative sequences (one per patient) is shown here. Figures were created using Jalview [18]; the presence of N-terminal glycosylation motifs is shown in yellow.

Fig 1 exemplifies an instance where hypervariability causes problems with an alignment of the HIV-1 envelope protein (ENV, also known as gp120) [5]. Two sequence properties of this viral protein family are key to its function. Firstly, it contains several ‘variable’ regions that are hypermutated to avoid detection by the host’s immune system. Secondly, *in vivo*, gp120 is richly decorated with glycans, hence N-linked glycosylation motifs are abundant in the sequence. The alignment in Fig 1 is generated using the state-of-the-art Clustal Omega [6] program; one can appreciate in the overview at the bottom that in general it does a great job, certainly in the constant regions; one, C3, is shown in detail as an example. (Note that the full alignment contains over a hundred sequences, but here we show a representative subset for clarity.) However, focusing on the variable regions (marked in red) with the glycosylation motifs (in yellow), it is equally obvious that these are generally poorly aligned. The detailed illustration of V1 shows that many of the motifs in this region are not aligned (Fig 1 top left). This is a typical case, which can be seen in many sequence data sets. In this gp120 study, the solution was to redress the misaligned regions by hand, which took the better part of two weeks to complete to satisfaction. We will return to the HIV ENV use case as an example in the results section.

As is the case with HIV ENV, the presence of motifs in a sequence may provide important clues about the function of that particular protein. Column-wise analysis of motif positions in a MSA may reveal information about motif conservation, implying selective pressure, which in turn suggests a functional role. This also means it is possible to use motif conservation across species to filter out motifs occurring by chance, only considering the motifs that are likely biologically active [7]. Obtaining a more accurate alignment through the inclusion of motif information will further help many downstream analyses; e.g. mutation impact scoring, residue specificity prediction or phylogenetic analysis.

We aim to tackle the motif alignment problem through our novel multiple sequence alignment strategy, Motif-Aware PRALINE (MA-PRALINE). MA-PRALINE receives motif patterns in PROSITE pattern syntax, matches them against the input sequences and biases the substitution scoring towards giving the motifs greater significance. This means that MA-PRALINE is not a *de novo* motif identification method. Motif patterns with significant matches in an input should first be identified through other means; for example, by database searching or running a motif discovery program. The strength of the bias towards motif alignment is controlled by a parameter, *α*. Larger values of *α* result in a stronger bias towards motif alignment, whereas *α* = 0 is equivalent to normal sequence alignment.

MA-PRALINE has been implemented on top of the existing multiple alignment program PRALINE [8]. PRALINE is a popular multiple alignment toolbox, with existing functionality to improve alignment quality by incorporating information about transmembrane regions (TM-PRALINE) [9], homology (PSI-PRALINE) [10] and secondary structure [11]. Key to the motif-aware alignment algorithm is the support for multiple sequence tracks in PRALINE; these tracks can contain multiple sources of data for every sequence position. Other sequence data could thus be incorporated in a similar manner, such as information about membrane-spanning segments or secondary structure.

Several related approaches to improve alignment quality have been attempted in the past. Db-Clustal [12] uses highly conserved fragments of sequences as anchor points to improve the quality of a multiple sequence alignment. COBALT [13] anchors the alignment using a consistent subset of constraints derived from domain information or from PROSITE [14] patterns. FMALIGN [15] allows the user to specify special conserved regions. These regions are then fixed in the resulting alignment; it is also possible to identify new conserved regions in an iterative manner. A key difference in the approach taken by MA-PRALINE, as opposed to these other methods, is the use of soft constraints. By assigning a score bonus, rather than restricting or anchoring the alignment, problems with false positives or spurious motifs can be mitigated more effectively.

In this work, we first developed a motif-aware alignment method. Secondly, we show, through a benchmark, that there exists a range of *α* values where motif information optimizes the alignment of motif-rich regions, while not compromising the overall alignment quality. We further validate our method by deriving an estimate of the motif conservation signal on another data set of reference alignments. We find that these two, largely orthogonal, estimates of the permitted range of *α* are in agreement. Finally, we illustrate the advantages of using a motif-aware alignment strategy, by considering the nitrate reductase, HIV ENV, cupredoxin protein families, all three containing conserved functional motifs.

Previously, we have shown that a similar approach is useful for aligning transcription factor binding motifs in DNA sequence regions [7]. In this work we show that a motif-aware approach can be equally useful for protein sequence motifs. To demonstrate MA-PRALINE in a practical context, we explore a number of real-world use cases. These include several difficult families from the alignment benchmark BAliBASE, as well as the HIV gp120 use case [5] introduced above. MA-PRALINE is available for Windows, Mac and Linux systems and, as open source software, can be found on GitHub at https://github.com/ibivu/MA-PRALINE.

## Materials and methods

Multiple sequence alignment (MSA) programs, such as PRALINE, Clustal Omega and others [6, 10, 11, 16, 17], take two or more sequences as input and return a sequence alignment as output. An alignment can be seen as an evolutionary superposition of biological sequences; it shows which symbols in the sequences are considered equivalent and which symbols have no counterpart in other sequences. (These unmatched symbols cause *gaps* in the other sequences.) Because extending the pairwise alignment algorithm to multiple alignment becomes computationally infeasible as the number of sequences grows, a heuristic called progressive alignment is commonly used [19, 20]. Progressive alignment grows a MSA by iteratively adding sequences to it through pairwise alignment. It is considered a reasonable heuristic, owing to the hierarchical nature of evolution; however, due to its nature as a greedy algorithm it is quite sensitive to the order in which the sequences are added to the alignment [21] (also known as the *join order*). PRALINE offers multiple facilities to mitigate the effects of this greediness: by incorporating information about all input sequences into *preprofiles*; and by determining the join order while the algorithm is growing the alignment instead of up front.

In addition to the standard alignment inputs, Motif-Aware PRALINE (MA-PRALINE) allows an end user to specify motif patterns in PROSITE pattern syntax [14]. This information is used to grant a bonus to the amino acid substitution score when matching two amino acids from the same motif, with the aim of improving the biological significance of the resulting alignment.

### Multiple sequence alignment strategy

Motif information is included in the pairwise alignment step of the algorithm. Multiple sequence alignment is performed using the progressive multiple alignment strategy on a pre-generated guide tree. The scores used to generate the tree dictating the join order are obtained by global pairwise alignment. The merging step of the progressive multiple alignment algorithm is also performed by global alignment of blocks. This strategy, while considerably less advanced than the standard PRALINE strategy (creating a guide tree on the fly), was chosen because it performed well in our earlier work on motif alignment [7]. It should also be noted that MA-PRALINE can be configured to use the full suite of enhancements that regular PRALINE employs to increase the alignment quality. Information about the run time performance of MA-PRALINE is given in S1 Appendix.

To generate the multiple sequence alignment a pairwise score matrix *P* is first obtained by aligning all pairs of input sequences. This matrix is then transformed into a dissimilarity matrix by shifting the values such that the highest scoring pair receives the value of zero and the lowest scoring pair receives the value of −min(*P*) + max(*P*).

$$D_{xy}=D_{yx}=-P_{xy}+\max\left(P\right)$$ (1)

Here *D*_*xy*_ is the dissimilarity between sequences *x* and *y*; *P*_*xy*_ is the alignment score between input sequences *x* and *y*. A guide tree is subsequently built from the full dissimilarity matrix *D* through hierarchical clustering. The linkage method is set to UPGMA by default, but single and complete linkage are also available as an option. Finally, a multiple sequence alignment is extended by pairwise alignment in the order given by the guide tree. The PRALINE alignment method [8] was adapted for this work to perform the motif-aware multiple sequence alignment, as further detailed in the next section.

### Motif-aware pairwise alignment

In the progressive multiple alignment strategy a multiple sequence alignment is grown iteratively by merging new sequences into it by pairwise alignment. In PRALINE these pairwise alignments are performed with a dynamic programming algorithm; both global (Needleman-Wunsch [22]) and semi-global (PRALINE and others) merge strategies are supported. The algorithm guarantees that the optimal alignment will be found. This optimality is defined as the most probable alignment according to the provided probability model. This model describes the likelihood of changing one kind of symbol (such as a type of amino acid) into another kind or into a gap. Inserting a gap means that a symbol in one of the two sequences has no corresponding symbol in the other.

The algorithm works in two steps. In the first (or forward) step the solution matrix *F* is obtained by iteratively solving a recursive equation. In the second (or traceback) step a maximally scoring path is reconstructed from *F*. This path then corresponds to (one of) the most probable alignment(s) between the two sequences. In the global strategy the path is restricted to start in the bottom right corner of *F* and to end in the top left corner of *F*. In the semi-global strategy this requirement is relaxed: paths may start in the maximally scoring cell in the last row or column of *F* and may end in any cell of the first row or column. This has the effect of making gaps at the beginning and end of an alignment free, which in turn improves the quality of an alignment between sequences of strongly varying lengths.

Information about the presence of one or more motifs is incorporated into this standard algorithm through the addition of a scoring term to the general recursive equation used to calculate the dynamic programming matrix *F*. Alternatively, one may think of this as extending the alphabet of the sequence (and the substitution matrix) to account for the possible presence of a motif [7]. The formulation of the modified recurrence relation is given below. Note that this is a general definition; MA-PRALINE additionally implements an optimized version that improves the execution time at the cost of restricting the gap penalty function *g*(*l*) to be linear (*g*(*l*) = *dl*) or affine (*g*(*l*) = *e* + *d*(*l* − 1)).

$$F_{ij}=\max\{\begin{array}{cc} F_{k,j}+g\left(i-k\right) & \text{for}\;k=0,...,i-1\\ F_{i,k}+g\left(j-k\right) & \text{for}\;k=0,...,j-1\\ F_{i-1,j-1}+S\left(A_{i},B_{j}\right)+S_{\text{motif}}\left(A_{i},B_{j}\right) \end{array}$$ (2)

*F*_*ij*_ is the value of the dynamic programming matrix *F* in row *i* and column *j*, *g*(*l*) is the gap penalty associated with a gap of length *l*, and *S*(*A*_*i*_, *B*_*j*_) is the match score between the symbols at position *i* and *j* in sequences *A* and *B*. *S*_motif_(*A*_*i*_, *B*_*j*_) is the motif scoring term, defined as follows.

$$S_{\text{motif}}\left(x,y\right)=\{\begin{array}{cc} \alpha & \text{if}\;\text{both}\;x\;\text{and}\;y\;\text{are}\;\text{part}\;\text{of}\;\text{the}\;\text{same}\;\text{motif}\\ 0 & \text{otherwise} \end{array}$$ (3)

**Fig 2:**
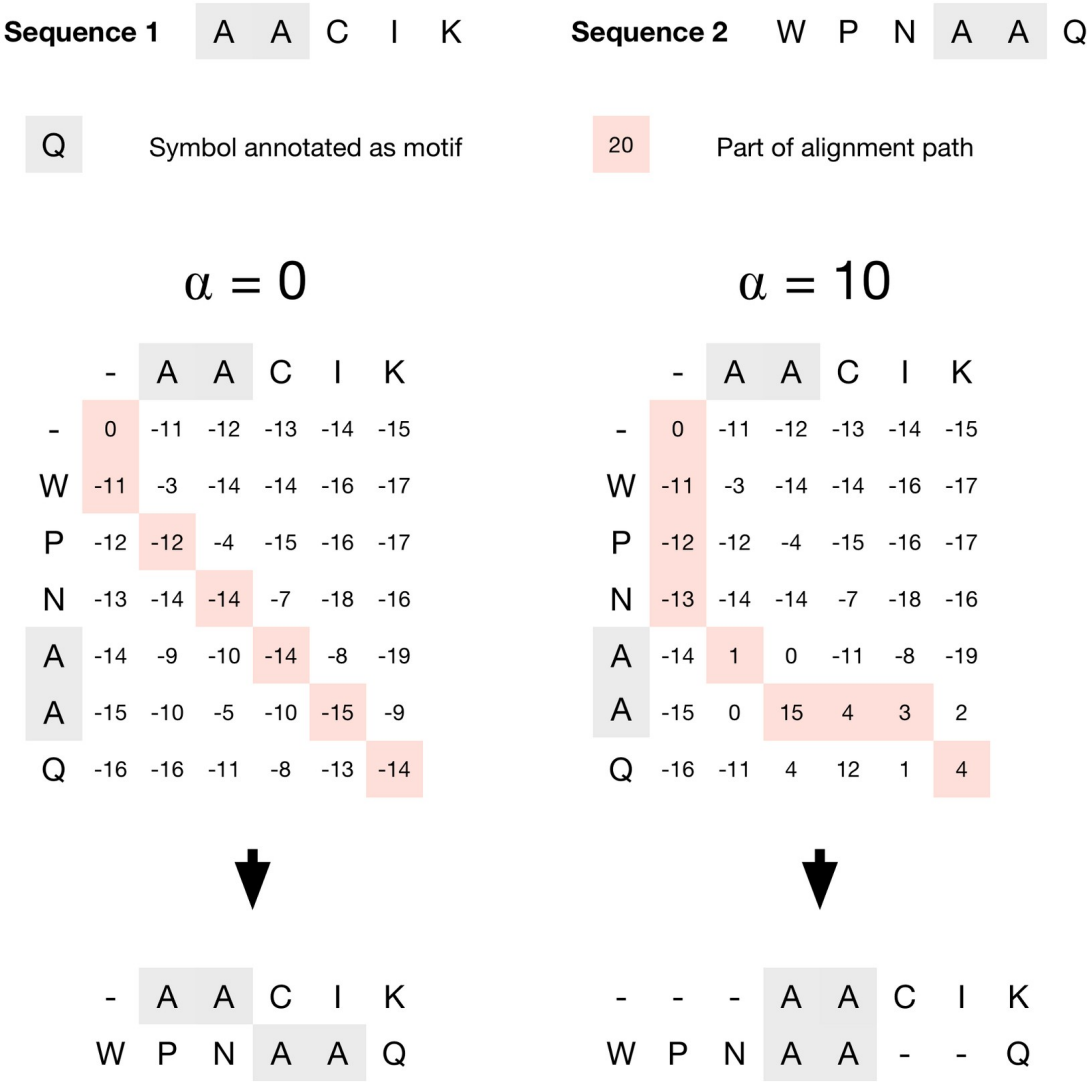
Example showing the influence of *α* on an alignment. Positions shaded in gray show amino acids which are part of a motif pattern match (the pattern *A-A* in this example). The path taken through the dynamic programming matrix is shown shaded in red. BLOSUM62 was used to score amino acid substitutions, together with a gap open penalty of −11 and a gap extension penalty of −1 (or, *g*(*l*) = −11 + (−1 * (*l* − 1)) for *l* > 0 and *g*(*l*) = 0 for *l* = 0).

*α* is a parameter that controls the strength of the bias towards motif alignment. If *α* is set to a large value, the algorithm will have a very strong preference to align motifs over the maximization of the traditional amino acid substitution score. In the case of *α* = 0 the behavior reverts to that of the conventional dynamic programming alignment algorithm. An example showing the influence of *α* on an alignment is shown in Fig 2.

### Motif annotation

Protein motif annotations are provided to the program in the form of PROSITE regular expression pattern definitions. Patterns in this format can be found in a number of places, such as in PROSITE [14, 23, 24] itself and in the Eukaryotic Linear Motif (ELM) [25] database. If the structural family of a set of proteins is known, it could be possible to include structural motifs from a database like SMoS [26]. Finally, because the PROSITE pattern format is simple and widely used, it is also possible for an end user to encode a previously undocumented pattern by hand.

PROSITE pattern syntax is an example of a regular expression (or *regex* in short). Regular expressions are a compact way to encode a sequence pattern in a manner that allows for efficient searches through large biological databases. For example, the N-linked glycosylation motif introduced earlier is encoded by the PROSITE pattern *N*-{*P*}-*[ST]*-{*P*}. This pattern matches any subsequence of amino acids starting with an asparagine (*N*), followed by any amino acid but a proline (*P*), followed by either a serine (*S*) or a threonine (*T*) and terminated by any amino acid but a proline (*P*).

**Fig 3:**
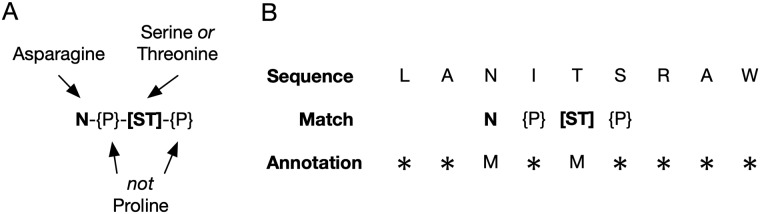
An explanation of the motif matching rules and the motif annotation process. A: N-terminal glycosylation site motif pattern, with an explanation of its match rules. Informative match rules are shown in bold. B: Example sequence (top row) matched against the N-terminal glycosylation site pattern (middle row). The bottom row shows whether a position is annotated as a motif match (*M*) or not (*).

The motifs are used to annotate matching regions of input sequences. Motif patterns, however, often contain information about the spacing between matching symbols. For example, the pattern *N-x(8)-N* can be read as “match any sequence consisting of two asparagines separated by 8 amino acids of any type”. Here, only the two asparagine positions can reasonably be considered part of the motif; the other positions merely offer information about spacing. We thus only consider a position as part of a motif if it matches against an informative rule in a pattern. A rule in a pattern is considered informative if it excludes more types of amino acids from matching than it includes. For example, the rule “match any amino acid that is not proline” is uninformative because it only excludes one type from matching. However, the rule “match either alanine or tryptophan” is informative because it excludes all but two types. A typical PROSITE motif pattern is described in Fig 3, together with an example sequence matched against it. If the above set of rules erroneously considers a motif position as uninformative, MA-PRALINE can be configured to treat all motif matches as informative. This may especially be desirable in the case of motif patterns which are too short to contain information about spacing. More information regarding the different ways in which motif information can be provided to MA-PRALINE is given in the Supporting Information, S2 Appendix.

### Fitting α* on a reference data set

In order to obtain an orthogonal estimate of allowed values for the motif score parameter *α*, we apply a knowledge-based approach on a reference data set. The methodology used is similar to the way BLOSUM matrices [3] are determined. HOMSTRAD was chosen as the reference because it is strictly based on structural alignments. In the other reference sets (such as BAliBASE), expert manual adjustments to the sequence alignment may have introduced a bias towards aligning known motifs.

To construct our input set, all reference alignments in HOMSTRAD are annotated with all of the motif patterns present in PROSITE. This yields gapped alignments with both an amino acid sequence and one or more motif annotations. Reference alignments without a single motif match are excluded from the input set. Using this procedure, we find 34568 motif matches in 3102 sequences, over a total of 974 HOMSTRAD alignments. The minimum, maximum and mean amount of matches per alignment are 0, 619 and 35.49, respectively. Per sequence there is a minimum number of matches of 0, a maximum of 69 and a mean of 11.14. Additional statistics regarding motif redundancy in HOMSTRAD are given in S3 Appendix.

We define α* as the logarithm of the probability of observing the alignment of a motif annotated residue, divided by the expected (or naive) probability of such an event. In other words, α* expresses how many times more likely the alignment of motifs is than would be expected purely by chance. α* is given by the logarithm of the ratio between the observed motif alignment probability *P*(*O*_motif_) and the expected, or background, probability *P*(*E*_motif_).

$$\alpha^{*}=f\log_{b}(\frac{P(O_{\text{motif}})}{P(E_{\text{motif}})})$$ (4)

The scaling factor *f* = 2 and logarithm base *b* = 2 are chosen so that the parameter scale is equivalent to that of standard BLOSUM matrices [3], and to that of *α*. This allows for direct comparison between values of *α* and α*. In order to obtain *P*(*O*_motif_) we count, for every column in an alignment, the number of symbol pairs in which both symbols are a match symbol. The probability then becomes this frequency divided by the total number of pairs over all columns in the same alignment. If an alignment contains gaps, then these are treated as a non-match symbol. The expected probability *P*(*E*_motif_) is estimated by the square of the fraction of positions containing match symbols (*q*_*m*_^2^). This estimate is reasonable as long as motifs are distributed evenly across the different sequences in an alignment.

### Scoring

The quality of an MSA is measured in terms of the Sum-of-Pairs score (SP score) [27–29] versus a reference alignment. The motif score is defined as the number of motif annotated symbol pairs in an alignment column, divided by the total amount of pairs. The motif score is counted over all columns in an alignment containing at least a single motif match. If multiple patterns are included, their matches are collapsed into a single motif annotation. Consequently, if a certain sequence position matches multiple patterns the pairs are only contributing to the motif score once.

### BAliBASE benchmark set

BAliBASE [30] contains a large number of manually curated reference alignments for the purpose of benchmarking MSA programs; for an in-depth discussion of the difference between the sets we refer to earlier work [29]. The BAliBASE reference alignments and program outputs were used as-is; no changes were made by hand.

All families with significantly matching motifs in the most recent release of BAliBASE 4 were used to assess motif and overall alignment quality for various MA-PRALINE *α* values—please see S3 Table. for a full list of families and the motif patterns that were annotated in them.

### Use cases

We will discuss two families from BAliBASE 3, BB20035 and BB30015, and one HIV envelope protein family, in more detail. These represent typical examples of difficult to align sequence families with known functional motifs. For the alignments in the results section we use alignment parameters that one would typically use for these specific families, as described below. Note that non-default parameter choices—the substitution matrix, semiglobal merging and the use of preprofiles—do not affect the quality of the aligned motif regions. A full overview of the parameters is given in S4 Appendix.

For the two BAliBASE use cases the additional alignment settings that were used to generate the MA-PRALINE alignment are: semiglobal alignment merging, because the sequences vary greatly in size; the BLOSUM40 and BLOSUM30 substitution matrices, because a lower-than average sequence conservation is expected in these families; and finally global preprofiles, because this is a commonly enabled quality improvement, which only incurs a mild penalty in terms of run time.

#### Use case: BAliBASE 3 alignment set BB30015

This sequence set consists of two major families of copper protein, namely the cupredoxin and nitrous-oxide reductase families. Cupredoxins are copper binding electron transport proteins that are commonly involved in photosynthesis and respiration pathways [31]. The main domain of the cupredoxins consists of a beta-sandwich domain which supports a copper binding site (S1 Table). Typical structural features present in the plastocyanin and azurin subgroups are a ‘kink’ in the C-terminal beta-sheet (shown in magenta in S1 Fig) and a ‘tyrosine loop’. Both features are a possible consequence of the beta-sandwich structure. The major difference between the two subfamilies is the number of loops in the protein—azurins are comprised of five loops while plastocyanins only have four [31]. The plastocyanin and azurin subfamilies are notoriously difficult to align, even though they are homologous [8].

Nitrous-oxide reductases are bacterial homodimeric enzymes that catalyze the reduction of nitrous oxide (N_2_O) to dinitrogen (N_2_). Each chain of the homodimer consists of two domains, namely an N-terminal 7-bladed beta-propeller domain containing the catalytic site (CU_Z_ domain) and a C-terminal cupredoxin-like domain (CU_A_ domain) [32] (S2 Fig). Although the CU_A_ domain is structurally similar to cupredoxin proteins, it does not share the copper binding motif.

#### Use case: BAliBASE 3 alignment set BB20035

This sequence set belongs to reference group 2, which contains alignments of protein families with a number of orphan proteins—or proteins that do not have detectable homology at the sequence level to the other members of a particular set [33]. The majority of the sequences in BB20035 are nitrate reductases, a type of molybdoenzyme whose function is the catalysis of nitrate to nitrite. These proteins are commonly involved in nitrate assimilation pathways in all kingdoms of life. According to the corresponding entries in the UniProt database [34], the nitrate reductases in the BB20035 set possess a cofactor binding site for: heme, molybdopterin and flavin adenine dinucleotide (FAD) (S1 Table).

#### Use case: HIV-1 envelope glycoproteins

The HIV-1 envelope glycoproteins (ENV) belong to a group of glycoproteins which are exposed on the surface of the HIV viral envelope. These proteins play an essential role in the ability of the virus to bind to the host cell receptors and thus to initiate infection. The glycosylation of certain regions in these envelope proteins is known to be important for the survival of the virus. The sugars attached by glycosylation act as some kind of shield, protecting the virus from detection and attack by the immune system of the host [5, 35, 36]. The settings used to generate the alignments of HIV-1 ENV, documented in S4 Appendix, are equivalent to the settings used for Table 1, with *α* = 20.

**Table 1:** Average benchmark SP and motif scores for various *α* over BAliBASE 4. The standard deviation for both the motif and SP scores is also given. For low values of *α* the motif score increases while the SP score remains stable. Past *α* = 15, however, the SP score starts to decrease rapidly. The standard deviation for both the motif and SP scores grows as *α* is increased.

α	Avg. SP	Std. SP	Avg. motif	Std. motif
0	0.664	0.188	0.048	0.054
5	0.668	0.186	0.049	0.054
10	0.661	0.188	0.052	0.057
15	0.646	0.189	0.055	0.059
20	0.630	0.191	0.057	0.060
25	0.603	0.197	0.060	0.062
30	0.576	0.202	0.062	0.063
35	0.554	0.201	0.065	0.064
40	0.528	0.201	0.069	0.067
45	0.502	0.199	0.072	0.069
50	0.480	0.202	0.075	0.071
100	0.341	0.190	0.093	0.077

ENV sequences found in different patients have a relatively high sequence similarity overall, but the N-terminal glycosylation site motifs (S1 Table), located in the flexible loop regions, tend to be highly variable. This makes these flexible regions particularly difficult to align properly using conventional alignment methods, as shown in a study by van den Kerkhof *et al*. [5].

## Results

We start by evaluating our method in a more quantitative fashion, considering both the classic Sum-of-Pairs score (SP score) [27–29] to assess the quality of the alignment, as well as the quality of the motif alignment through a motif SP score analog. In addition, we study how the value of the motif score parameter *α* influences the balance between the quality of the alignment of motifs and the overall alignment quality. Finally, by considering the HIV ENV, cupredoxin and nitrate reductase use cases, we will illustrate the benefits of our motif-aware alignment method for problems where it is crucial to have motifs correctly aligned.

### BAliBASE 4 benchmark set

In order to test performance and the effect of *α* on the overall alignment quality, we ran MA-PRALINE over a range of *α* values on alignments from BAliBASE 4 containing preserved motifs. Preserved motifs were identified by matching sequences against all of the motif patterns documented in PROSITE. A motif is considered to be preserved if at least 50% of the sequences in a BAliBASE alignment contain at least one instance of it. All of the 218 alignments in BAliBASE 4 have at least one motif that meets this threshold. In order to keep the computational run time feasible, 22 very large alignments were excluded from the benchmark set. For a full list of BAliBASE 4 alignments and the motif patterns that were used per alignment, please see S3 Table.

Table 1 shows the average SP and motif benchmark scores of alignments generated by MA-PRALINE using a range of *α* values. These results show that, for small values of *α*, the overall SP score remains stable while the motif score increases, indicating an improvement in the quality of the motif alignment. If *α* is increased past a limit of around 15, however, the overall quality starts to suffer. These findings show that it is possible to improve the quality of the motif alignment without strongly affecting the overall alignment quality. According to these results, *α* should be set between 5 and 20, depending on the degree of motif conservation. The standard deviation across different BAliBASE alignments also grows for both the SP score and the motif score; this may indicate that the inclusion of *α* is changing the order in which sequences are added to the alignment by the progressive alignment algorithm. Changes in join order may result in large changes to an alignment, since progressive multiple sequence alignment algorithms are very sensitive to the order in which sequences are added to the alignment.

### Estimation of the motif conservation signal, α*

**Fig 4:**
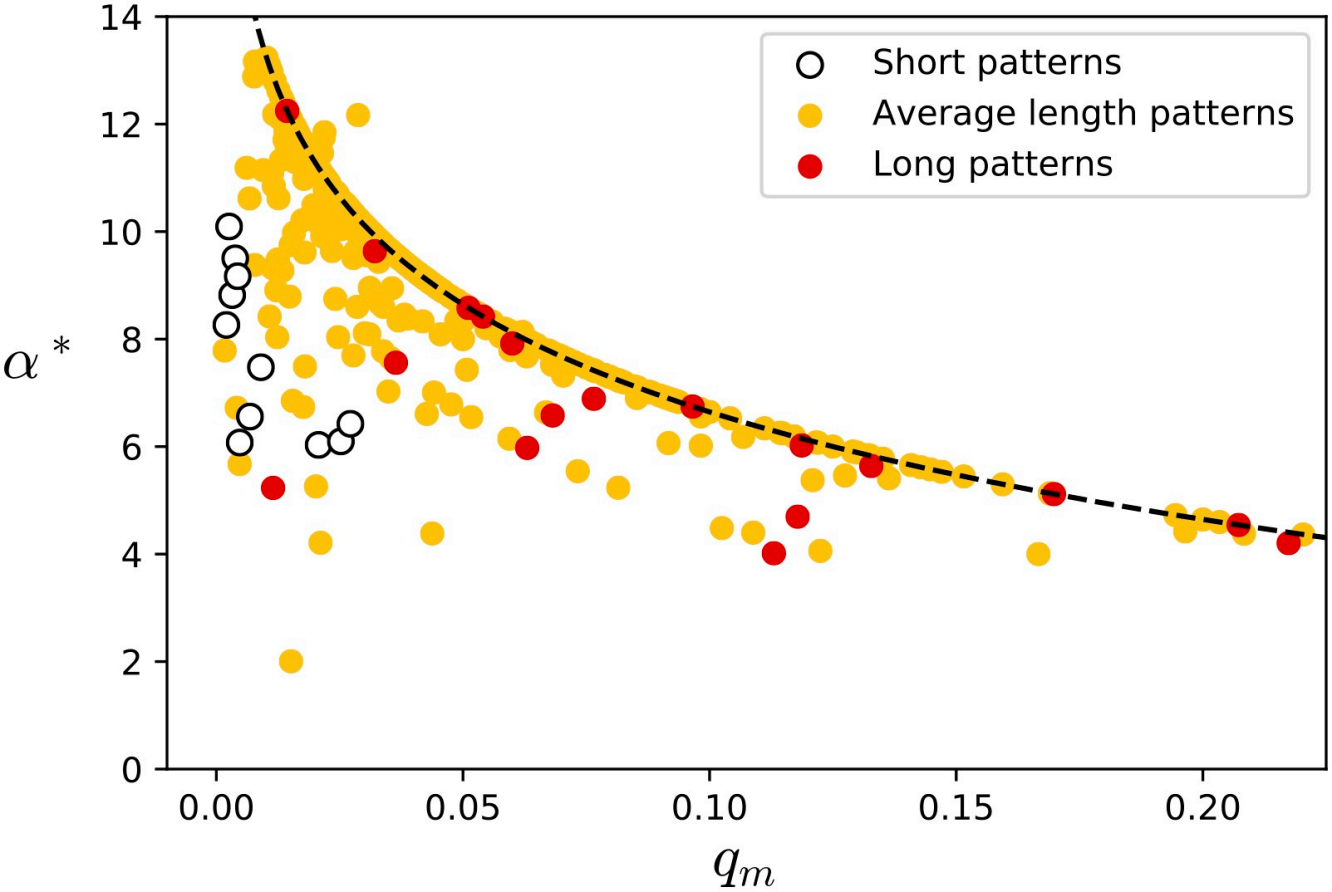
The estimate of the motif match score α*, derived from the HOMSTRAD set of reference alignments. Every point corresponds to a single motif pattern found in PROSITE that has at least one match in HOMSTRAD. *q*_*m*_ is the fraction of amino acids matching a pattern versus the total number of amino acids in all HOMSTRAD alignments containing at least one instance of the pattern. Short patterns are defined as having fewer than 6 match rules, average length patterns as having between 6 and 20, and long patterns as having more than 20. The dashed line indicates the α* limit for perfect motif conservation; the four points that most prominently fail to obey the limit correspond to PROSITE entries PS00022, PS00142, PS00370 and PS00589.

Given the viable range of *α* found by benchmarking MA-PRALINE (5 < *α* < 20), we want to see whether this corresponds to the motif conservation signal we observe in reference alignments. Statistically derived through a similar methodology as BLOSUM matrices [3], we obtain an estimate of the motif conservation signal, α*. The derivation of α* was calculated over the HOMSTRAD data set [37], because it contains fewer biases than BAliBASE. Fig 4 shows the value of α* for every motif pattern found in PROSITE with at least one match in HOMSTRAD. *q*_*m*_ is the fraction of amino acids which match a motif pattern versus the total number of amino acids in all sequence sets with at least a single match of the same pattern. Almost all values of α* fall within a decaying envelope imposed by *q*_*m*_; this is because α* is a measure of (logfold) additional conservation over the background probability. The scenario of perfect conservation, where every occurrence of a motif is aligned exclusively against other occurrences of the same motif, translates to an upper bound on α*. The bound is dependent on *q*_*m*_ because *q*_*m*_^2^ is used as the estimate of the background probability *P*(*E*). This is roughly analogous to the reason why rare amino acid types generally receive higher scores in a BLOSUM matrix. The data points which do fall outside of the envelope correspond to perfectly conserved motifs in HOMSTRAD; additionally, these patterns match multiple families of strongly varying sizes. In such cases *q*_*m*_^2^ underestimates the background probability, which in turn allows the α* value of these patterns to transgress the boundary. A list of all PROSITE patterns and associated *q*_*m*_, α* values is available in S4 Table.

Most of the longer motifs are of higher complexity, and thus have a lower chance of matching a subsequence spuriously. For these motifs it would be possible to choose a suitable value of *α* using *q*_*m*_ alone, which is known to the user in advance (after annotation). For the shorter (or lower complexity) motifs this does not seem to be true, however, with an observed range of α* between 6 and 10. Nonetheless, these results show a range of α* values largely in accordance with the acceptable range of *α*, independently obtained through the benchmark and the use cases. A value of *α* around 10 appears to be a reasonable setting, giving good alignment quality (as shown in Table 1) as well as being in accordance with the observed motif conservation signal for sequence evolution (as shown in Fig 4).

### HIV-1 envelope glycoprotein

The sequences of the HIV-1 envelope glycoprotein (ENV) contain many occurrences of the N-linked glycosylation motif (pattern *N*-{*P*}-*[ST]*-{*P*} with PROSITE identifier PS00001). Generating a globally acceptable alignment is not especially difficult as, outside of the variable regions, there is strong sequence conservation. The alignments produced by various alignment methods give very similar SP scores (S2 Table), indicating high alignment similarity. It is when a high-quality alignment for the variable regions is also required that one will run into difficulties; this is shown for a number of commonly used alignment programs in the Supporting Information (S8, S9 and S10 Figs).

**Fig 5:**
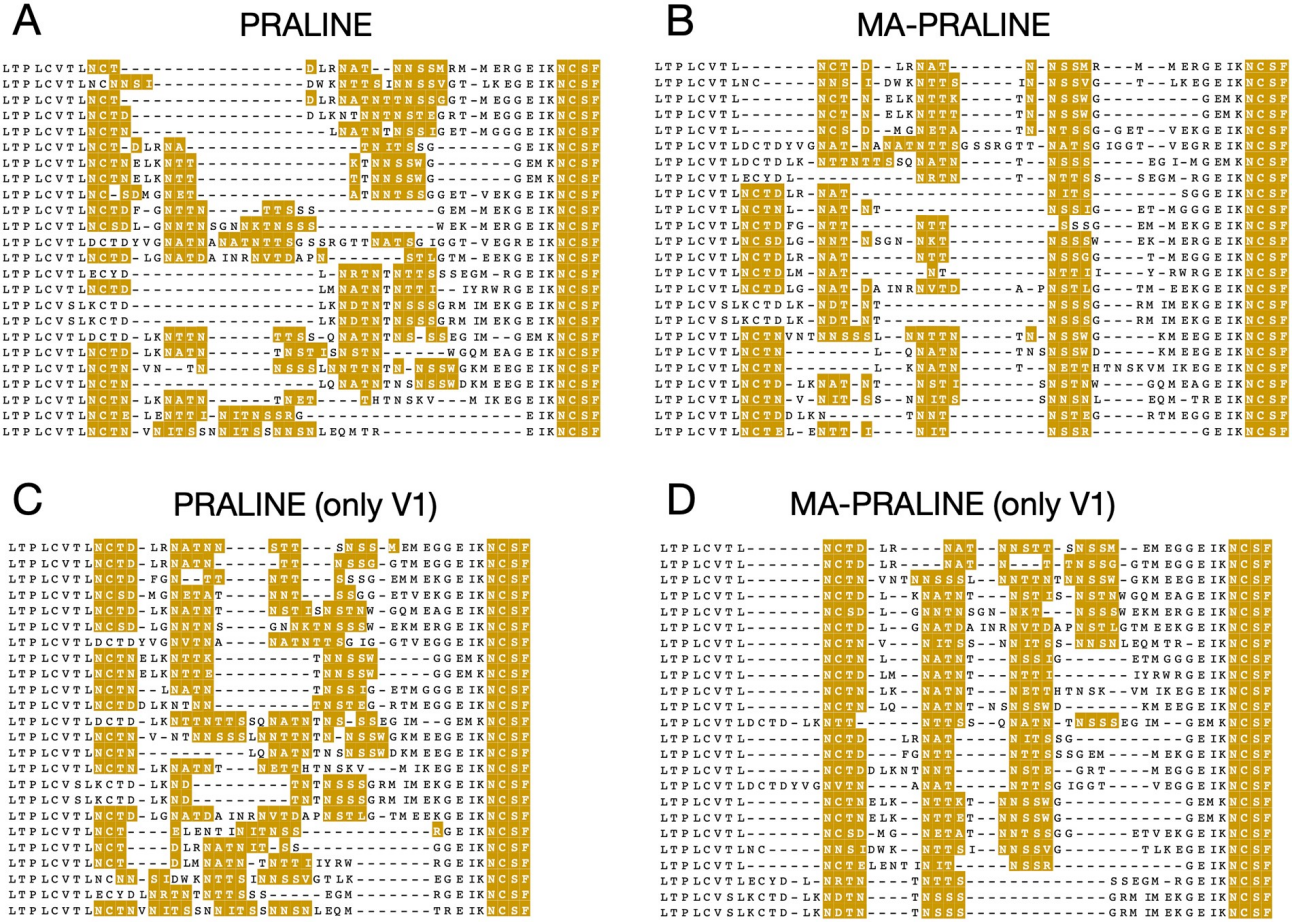
Incremental improvements for the difficult variable region V1 in the HIV ENV protein. Only the V1 region is shown in this figure; in cases where the full protein sequence was aligned, V1 was extracted afterwards using an alignment editor. Alignments were made using: A: regular PRALINE, aligning the entire protein; B: MA-PRALINE (*α* = 20), aligning the entire protein; C: regular PRALINE, aligning just V1; D: MA-PRALINE (*α* = 20), aligning just V1. Figures were created using Jalview [18]; the presence of N-terminal glycosylation motifs is shown in yellow.

In order to study the mechanism by which HIV evades an immune response, it is crucial to have a proper alignment of the glycosylation motifs, many of which are found in the variable regions [5]. Fig 5 shows how MA-PRALINE allows a user to optimize such alignments in various scenarios. When the full protein sequences are aligned, most MSA methods struggle to align the variable regions correctly, as shown in Fig 1 (for Clustal Omega) and in Fig 5, panel A (for regular PRALINE). With extra weight on the glycosylation motifs, MA-PRALINE is able to generate a reasonable alignment for these regions, as shown in Fig 5, panel B. If we only use the sequence region comprising V1 as input instead of the full sequence (and cutting V1 out afterwards), MA-PRALINE obtains even better results, as shown in Fig 5, panel D. Regular PRALINE still has difficulties (Fig 5, panel C).

#### Cupredoxin and nitrous-oxide reductase families (BB30015 alignment set)

**Fig 6:**
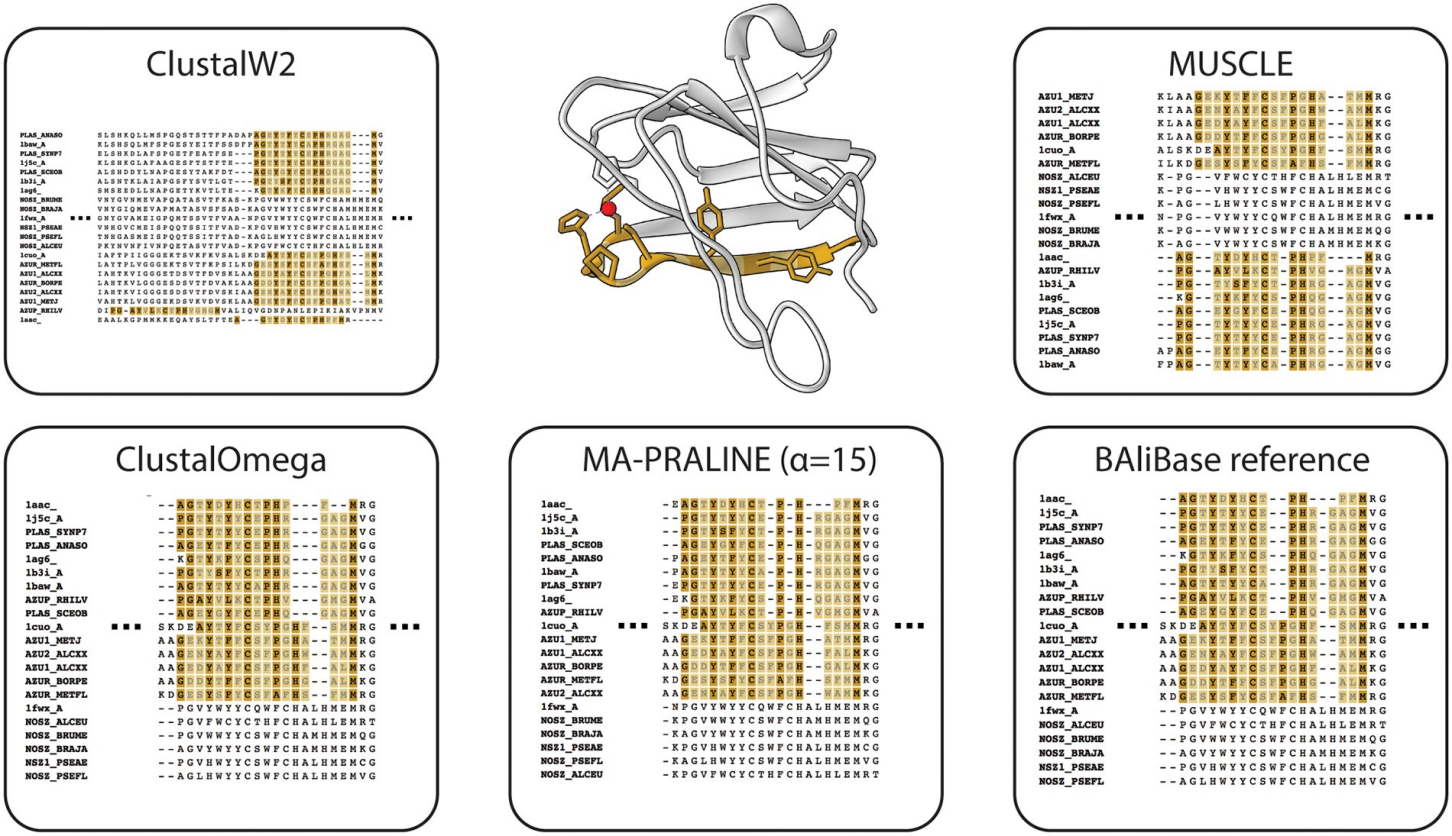
Alignment programs compared on the motif-rich region of the BAliBASE 3 reference alignment set of cupredoxin proteins (BB30015). Yellow colored residues match the copper binding motif (with PROSITE identifier PS00196). Lighter colors indicate non-informative residues within a motif match. Alongside the program output a reference structure is shown (PDB identifier 1AAC) to visualize the motif within the structural context of the protein family. Note that the proline residue at position 94 in the shown structure is misaligned in the BAliBASE reference. The colors used here are the same as in the alignment outputs. The ligand from the PDB structure is shown in red.

Fig 6 shows the aligned motif region of this protein family, together with a visualization of a reference structure. Note that even the BAliBASE reference contains a slight misalignment of a proline residue in the motif. This residue is an essential part of the copper binding domain, where it forms a ‘kink’ in the second beta sheet [31, 38] (middle panel Fig 6). Of the evaluated alignment programs only MA-PRALINE (with *α* = 15) produces a reasonable alignment of the motif, including the structurally important proline residue.

#### Nitrate reductase family with some orphan sequences (BB20035 alignment set)

**Fig 7:**
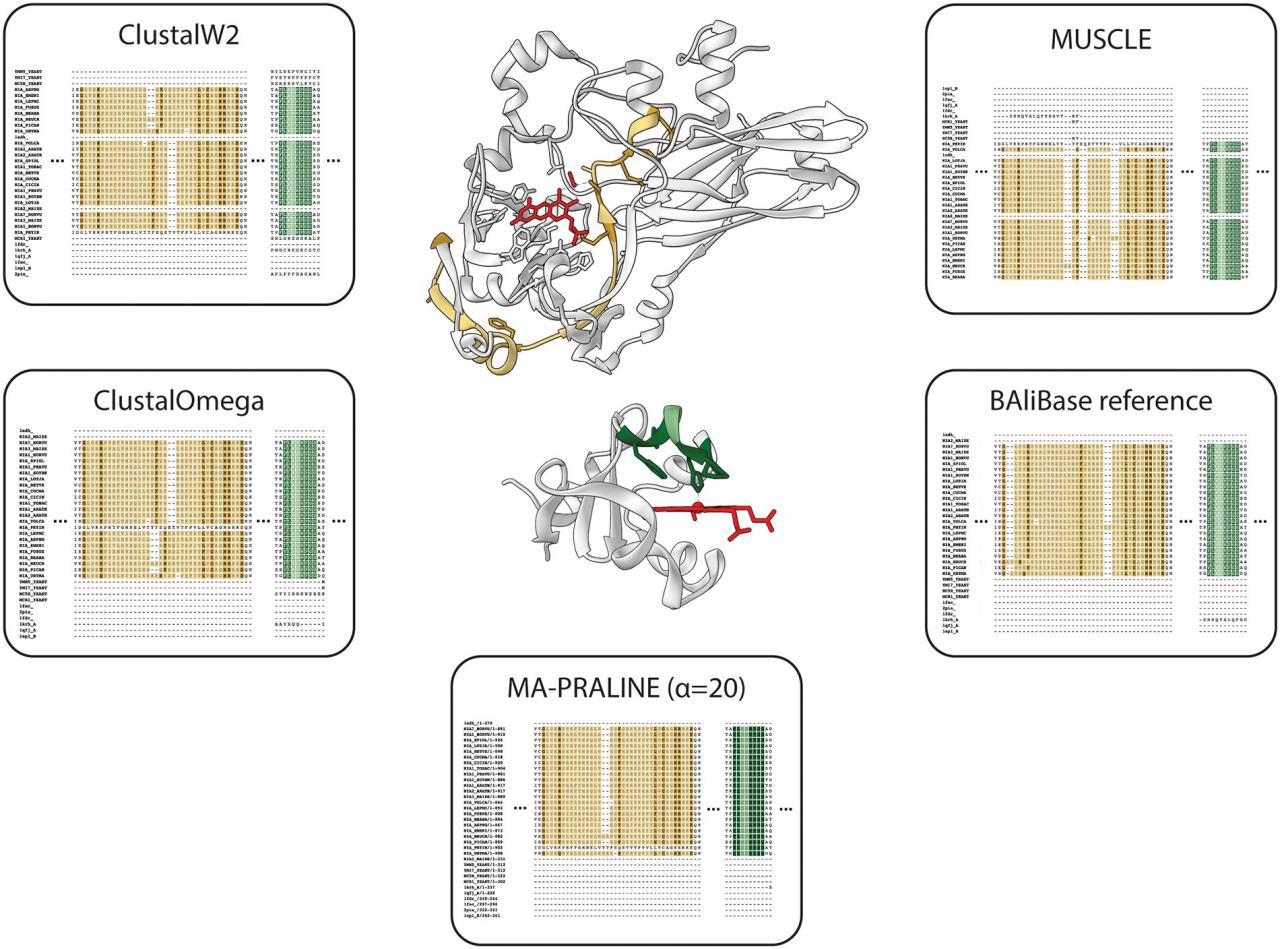
Alignment programs compared on the motif rich region of the BAliBASE 3 reference alignment set of nitrate reductase enzymes (BB20035). Residues colored yellow match against the eukaryotic molybdopterin oxidoreductase motif (PROSITE identifier PS00559). Residues colored green match against the cytochrome b5 heme-binding motif (PROSITE identifier PS00191). Lighter colors indicate non-informative residues within a motif match. Alongside the program output a reference structure is shown for each motif matching region (PDB identifiers: 2BIH for the oxidoreductase motif; 4B8N for the heme-binding motif) to visualize the motif within the structural context of the protein family. The colors used here are the same as in the alignment outputs. The ligand from the PDB structure is shown in red.

A comparison of different sequence alignment programs on the nitrate reductase use case is shown in Fig 7. Most regular MSA methods have difficulty with the eukaryotic molybdopterin oxidoreductase motif (depicted in yellow). MUSCLE appears to produce a slightly better alignment than Clustal Omega and Clustal W2, as it only misaligns one symbol of the motif. However, MUSCLE does insert too many gaps in the motif region. Only MA-PRALINE is able to align the motifs correctly.

## Discussion

MA-PRALINE is shown to perform well in aligning motifs in variable loop regions which are difficult to align using existing methods, exemplified by the HIV-1 envelope glycoprotein gp120 use case. Other viral proteins might benefit from alignment by MA-PRALINE, such as the glycoproteins which are involved in Hepatitis C cell binding and entry [39]. In general, this method could be used to improve parts of alignments for which the amino acid sequence is unstable but where motifs can be found to anchor the alignment. It should also be possible to use MA-PRALINE to improve the alignment of certain classes of proteins, which contain regions that are not strictly considered motifs, but where an additional conservation signal can be found. For example, one could apply the same principle on the membrane spanning regions of transmembrane proteins. This would provide another option for aligning membrane proteins, in addition to the existing TM-PRALINE strategy [9], which employs a transmembrane-specific residue exchange matrix.

Additionally, it should be noted that MA-PRALINE, in its current implementation, already supports nucleotide sequences. However, this study was done exclusively on protein sequences; any knowledge gained, such as supported parameter values, is not directly transferable to a nucleotide use case. For further information, we refer the reader to previous work [7], where an approach similar to MA-PRALINE was applied to DNA sequences.

### User recommendations

In this study we explored a number of real-life multiple sequence alignment use cases to demonstrate how MA-PRALINE could be used to improve the biological fidelity of a sequence alignment by aligning sequence motifs more accurately.

Motif alignment quality depends strongly on the value of the motif match score parameter (*α*). We also conclude that the correct value of *α* is dependent on the expected conservation of the motifs. Care should be taken not to harm the overall alignment quality by setting *α* to an unrealistically high value. A safe, default setting is *α* = 10, but depending on the sequence identity, the length of the motifs and possible motif redundancy, it may be possible to set *α* to a larger value. This may even be required to gain the full benefits of MA-PRALINE motif scoring. However, values larger than *α* = 30 lead to poor results. This *α* seems to indicate a threshold beyond which the motif boosting becomes strong enough to overcome the traditional substitution scoring for non-conserved motifs. We recommend trying out a few possible values within the range given above and to look at how the resulting alignment changes at each increment of *α*.

## Supporting information

S1 Appendix Performance figures of MA-PRALINE on variously sized inputs. (DOCX)

S2 Appendix Examples describing how to provide motif information to MA-PRALINE. (DOCX)

S3 Appendix Statistics regarding the redundancy of motif matches on the HOMSTRAD reference data set. (DOCX)

S4 Appendix Archive containing the inputs and alignments shown in the results. Also includes a script to generate the alignments with a local MA-PRALINE installation.
(ZIP)

S1 Fig The protein structure of the cupredoxin amicyanin, from Paracoccus denitrificans (PDB identifier: 1aac [40]). The copper binding motif is highlighted in yellow. Note the structural importance of the beta sheet kink induced by the proline at residue position 94, highlighted in magenta.
(TIF)

S2 Fig The crystal structure of nitrous-oxide reductase from Paracoccus denitrificans (PDB identifier: 1fwx [41]). Only chain A is shown. The cupredoxin-like domain (CU_A_) is colored according to its secondary structure.
(TIF)

S3 Fig The reference alignment of the BAliBASE 3 reference alignment set of nitrate reductase enzymes (BB30015). Colored residues are part of a motif match.
(TIF)

S4 Fig The reference alignment of the BAliBASE 3 reference alignment set of cupredoxins and nitrous-oxide reductases (BB20035). Colored residues are part of a motif match.
(TIF)

S5 Fig BAliBASE 4 benchmark performance plot. Reference based average SP and motif scores as a function of *α*.
(TIF)

S6 Fig BAliBASE 4 benchmark SP score box plot. (TIF)

S7 Fig Sections of the alignment of the HIV-1 envelope glycoprotein data set as produced by Motif-Aware PRALINE method, showing variable loop regions. The coloured residues indicate the location of the motif, semi-transparent colours indicate the spacer residues in the motif.
(TIF)

S8 Fig Sections of the alignment of the HIV-1 envelope glycoprotein data set as produced by Clustal Omega, showing variable loop regions. The coloured residues indicate the location of the motif, semi-transparent colours indicate the spacer residues in the motif.
(TIF)

S9 Fig Sections of the alignment of the HIV-1 envelope glycoprotein data set as produced by Clustal W2, showing variable loop regions. The coloured residues indicate the location of the motif, semi-transparent colours indicate the spacer residues in the motif.
(TIF)

S10 Fig Sections of the alignment of the HIV-1 envelope glycoprotein data set as produced by MUSCLE, showing variable loop regions. The coloured residues indicate the location of the motif, semi-transparent colours indicate the spacer residues in the motif.
(TIF)

S1 Table List of sequence motifs discussed in this study and their corresponding PROSITE pattern. (XLSX)

S2 Table Sum-of-Pairs alignment scores for the HIV-1 envelope glycoprotein data set. For MA-PRALINE two alignment strategies were evaluated: the canonical PRALINE on-the-fly strategy [11] and a simpler ‘pre-generated tree’ strategy.
(XLSX)

S3 Table List of BAliBASE 4 alignment sets containing one or more conserved motifs. BAliBASE identifiers are provided, along with matching motif patterns in PROSITE format.
(XLSX)

S4 Table Data used to generate Fig 4. A row corresponds to one point and lists the PROSITE accession, pattern, fraction of matching amino acids *q*_*m*_, and score α*.
(XLSX)

S5 Table Data used to generate Table 1. One row in the table contains the motif and SP scores for one pair of a BAliBASE family and a value of *α*.
(XLSX)
