# Supplementary material for: Motif-Aware PRALINE: Improving the alignment of motif regions
Source: PLoS Comput Biol. 2018 Nov 1;14(11):e1006547. doi: 10.1371/journal.pcbi.1006547 (PMC6233922; doi:10.1371/journal.pcbi.1006547)
Supplement: S1 Appendix — (DOCX) [file pcbi.1006547.s001.docx]

MA-PRALINE performance on inputs of varying sizes

Running on a single core of a recently released quad core Mac laptop, aligning the large HIV input file from the article takes 304 seconds and uses 740 MB of RAM. This input file contains 99 sequences of average length 826; a single motif pattern is annotated. If the same input set is taken, but with the sequences truncated after 410 amino acids, the run time decreases to 163 seconds with a peak memory usage of 330 MB. If, instead of halving the sequence length, the number of input sequences is halved (to 49), the run time and memory usage are 60 seconds and 272MB, respectively. Based on these performance figures, we expect the practical limit to be around 200 sequences of length 800.

In general, MA-PRALINE has a comparable runtime and memory footprint to 'normal' MSA programs. The dynamic programming algorithm for pairwise alignment has a complexity of O(mn), where m and n are the lengths of the two input sequences or profiles. The distance matrix generation has a complexity of O(n^2) pairwise alignments, where n is the number of input sequences.

MA-PRALINE can run individual pairwise alignments in parallel; given that the number of required pairwise alignments to generate the distance matrix is typically much greater than the number of cores, this results in speedups that are close to linear.
