# Supplementary material for: Motif-Aware PRALINE: Improving the alignment of motif regions
Source: PLoS Comput Biol. 2018 Nov 1;14(11):e1006547. doi: 10.1371/journal.pcbi.1006547 (PMC6233922; doi:10.1371/journal.pcbi.1006547)
Supplement: S2 Appendix — (DOCX) [file pcbi.1006547.s002.docx]

MA-PRALINE example usage

Motif information can be supplied to MA-PRALINE in two ways. The first is by providing a pattern in PROSITE syntax on the command line. The following command would annotate the pattern "N-{P}-[ST]-{P}" in all sequences and boost matches with an alpha of 5:

mapraline -p "N-{P}-[ST]-{P}":5 input.fasta output.aln

This should be sufficient for most use cases, but it is possible to provide the motif annotations directly if desired. In such a case MA-PRALINE will forego pattern matching entirely and simply load the annotation from a FASTA file. For example, assume a file named "input_motif.fasta" exists alongside the previously mentioned "input.fasta":

>seq1

*****MMMM*****

>seq2

**MMMM**MMMM*******

An asterisk means there is no motif match at a position while a capital M means there is a motif match at a position. A motif annotation FASTA file should contain sequences with the same sequence identifiers and lengths as the sequences in the input file; MA-PRALINE will give an error if this requirement is not met. The following command tells MA-PRALINE to load a motif annotation file and boost the scoring with an alpha of 10:

mapraline -a "input_motif.fasta":10 input.fasta output_manual.aln

Note that it is possible to mix the -p and -a options in a single invocation, with different values of alpha for each motif.
