# Supplementary material for: Motif-Aware PRALINE: Improving the alignment of motif regions
Source: PLoS Comput Biol. 2018 Nov 1;14(11):e1006547. doi: 10.1371/journal.pcbi.1006547 (PMC6233922; doi:10.1371/journal.pcbi.1006547)
Supplement: S3 Appendix — (DOCX) [file pcbi.1006547.s003.docx]

Motif redundancy in HOMSTRAD

In order to measure the redundancy of motif patterns matching sequences in HOMSTRAD we define a set of repeat matches as matches which are adjacent within *n* symbols. Two matches are adjacent in a sequence when the end of one match is followed by the start of another match in the set at most *n* symbols away.

For *n=1*, or the case in which matches are contiguous, repeat matches occur almost exclusively for short patterns, in particular the patterns N-{P}-[ST]-{P}, [ST]-x-[RK], [RK](2)-x-[ST], [ST]-x(2)-[DE], [RK]-x(2,3)-[DE]-x(2,3)-Y and G-{EDRKHPFYW}-x(2)-[STAGCN]-{P}. The minimum, maximum and mean length of a repeat set is 2, 12 and 2.12, respectively. Of the 34568 matches in total 5250 are repeats. If we relax the adjacency requirement to *n=5* the total number of repeats grows to 7880 and the mean increases to 2.24 matches per repeat set.
