# Supplementary figures and images for: Motif-Aware PRALINE: Improving the alignment of motif regions
Source: PLoS Comput Biol. 2018 Nov 1;14(11):e1006547. doi: 10.1371/journal.pcbi.1006547 (PMC6233922; doi:10.1371/journal.pcbi.1006547)

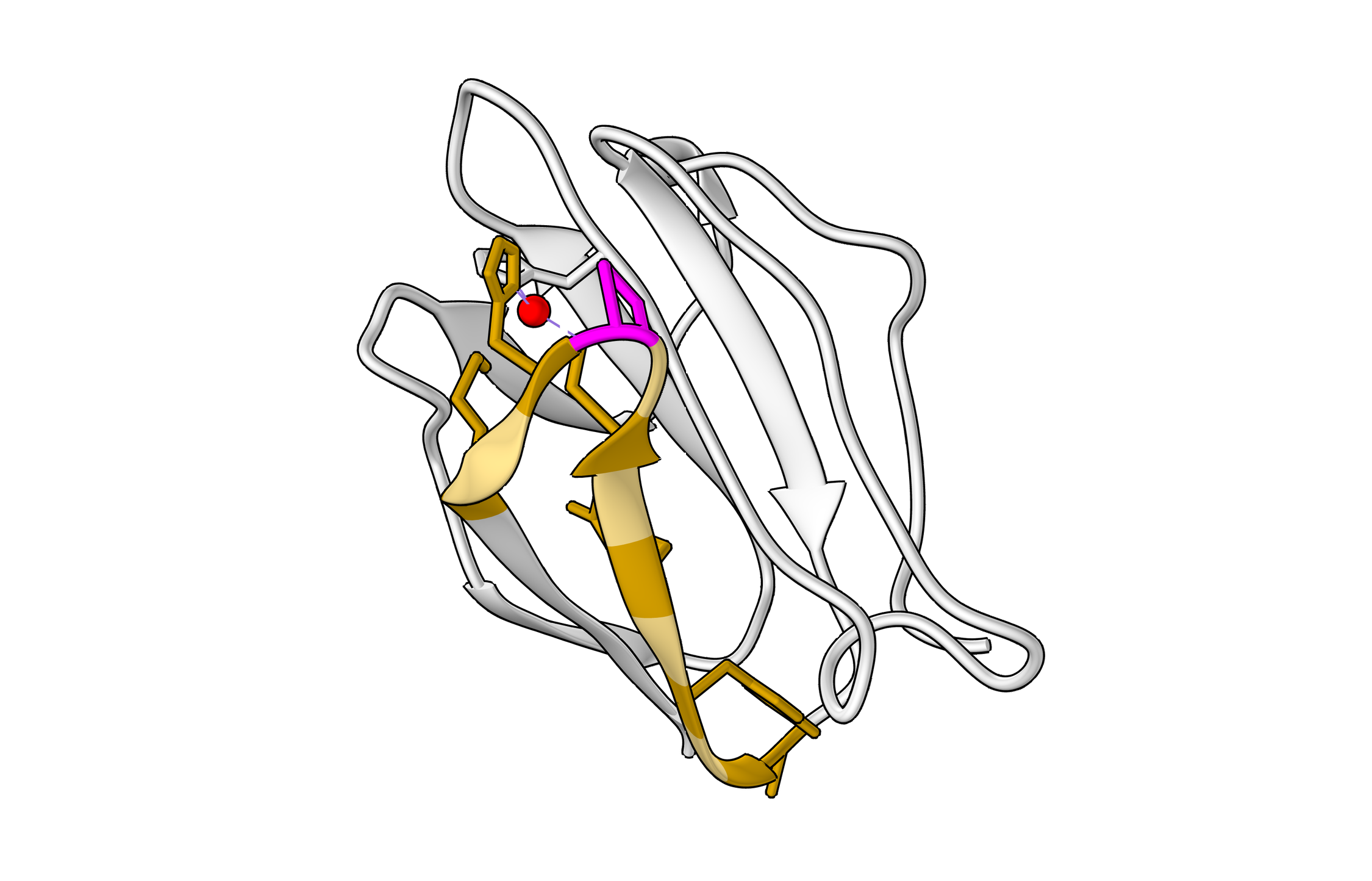

Supplement: S1 Fig — The copper binding motif is highlighted in yellow. Note the structural importance of the beta sheet kink induced by the proline at residue position 94, highlighted in magenta. (TIF) [file pcbi.1006547.s005.tif]

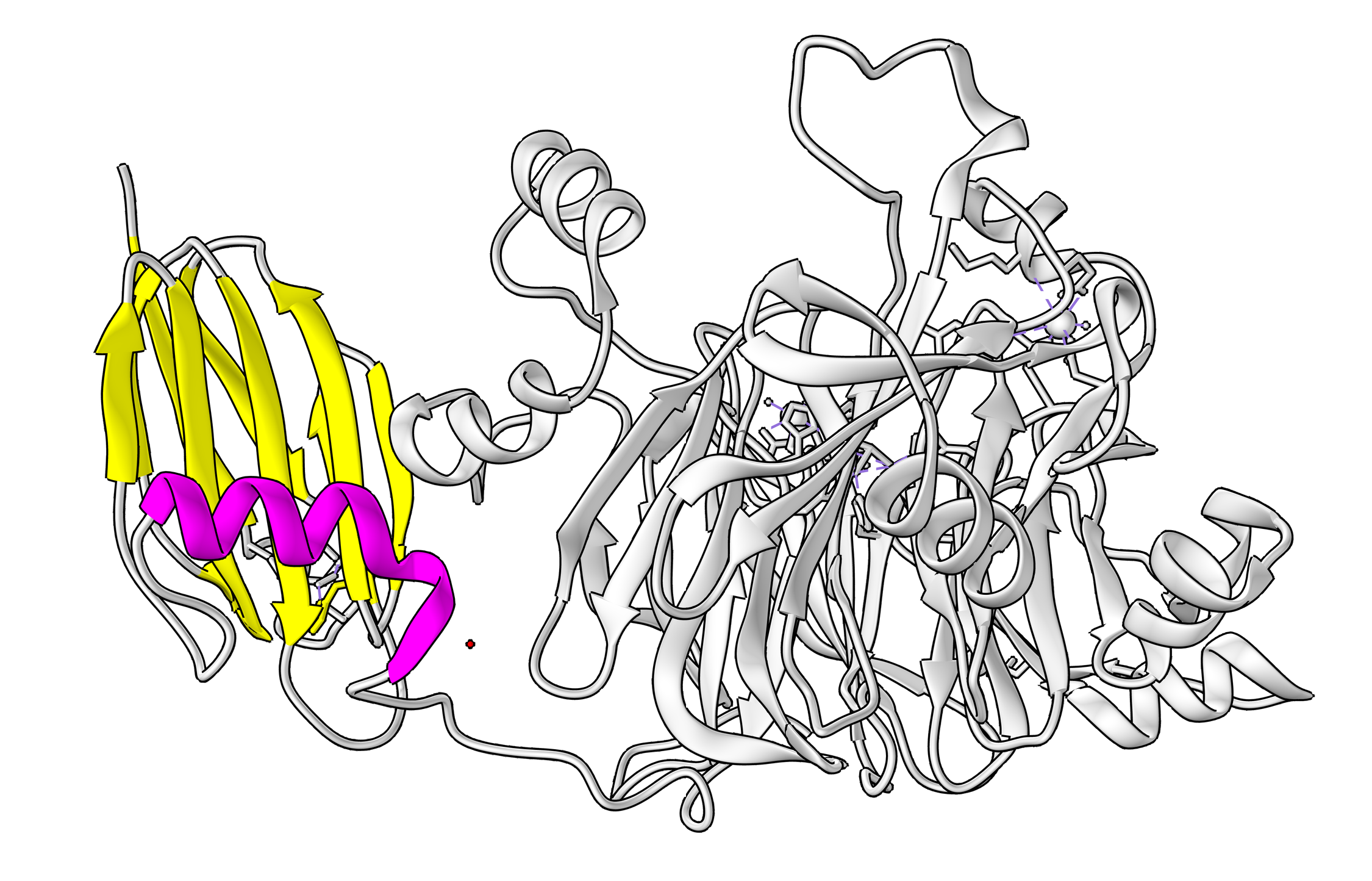

Supplement: S2 Fig — Only chain A is shown. The cupredoxin-like domain (CUA) is colored according to its secondary structure. (TIF) [file pcbi.1006547.s006.tif]

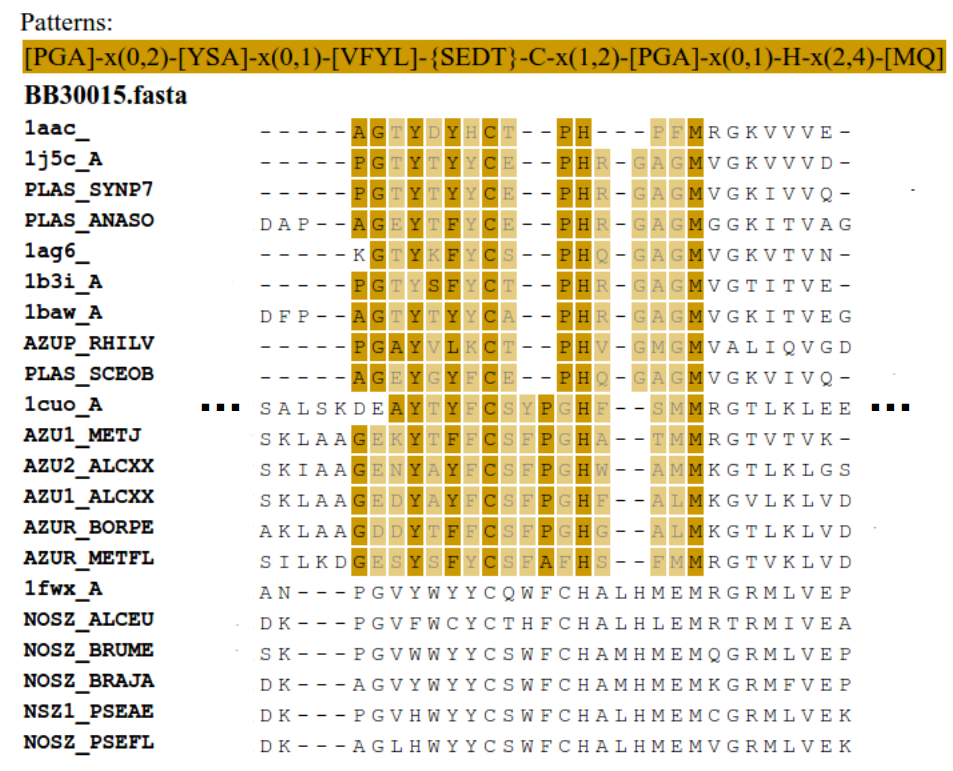

Supplement: S3 Fig — Colored residues are part of a motif match. (TIF) [file pcbi.1006547.s007.tif]

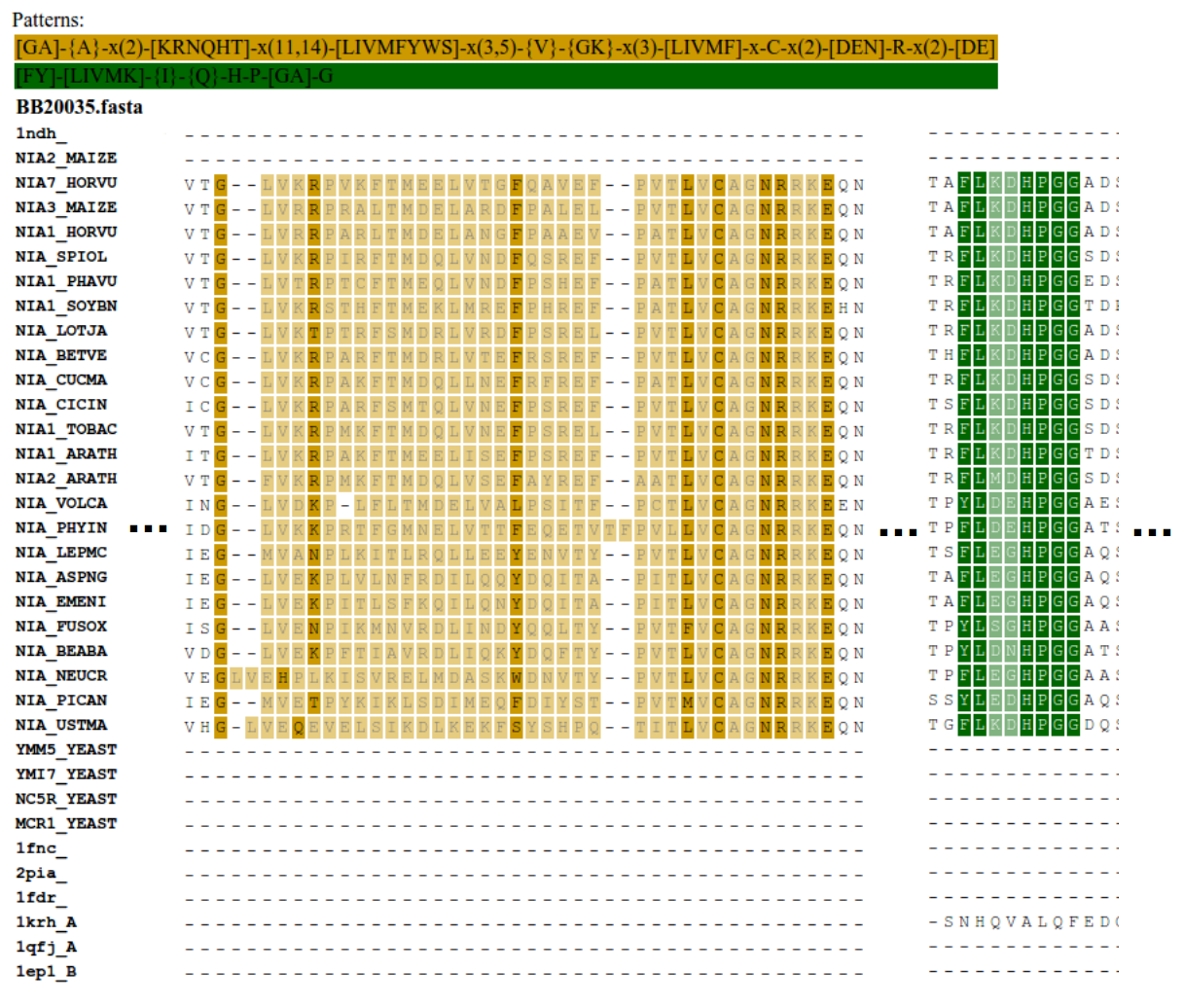

Supplement: S4 Fig — Colored residues are part of a motif match. (TIF) [file pcbi.1006547.s008.tif]

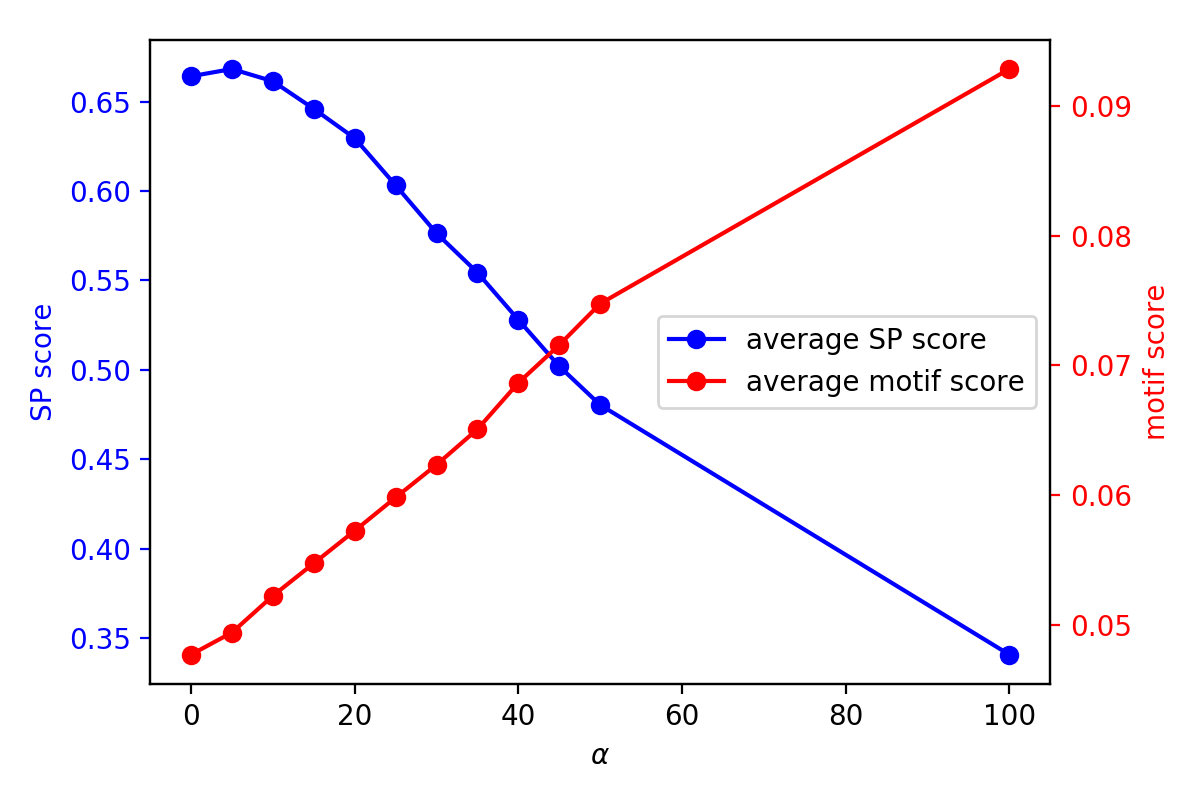

Supplement: S5 Fig — Reference based average SP and motif scores as a function of α. (TIF) [file pcbi.1006547.s009.tif]

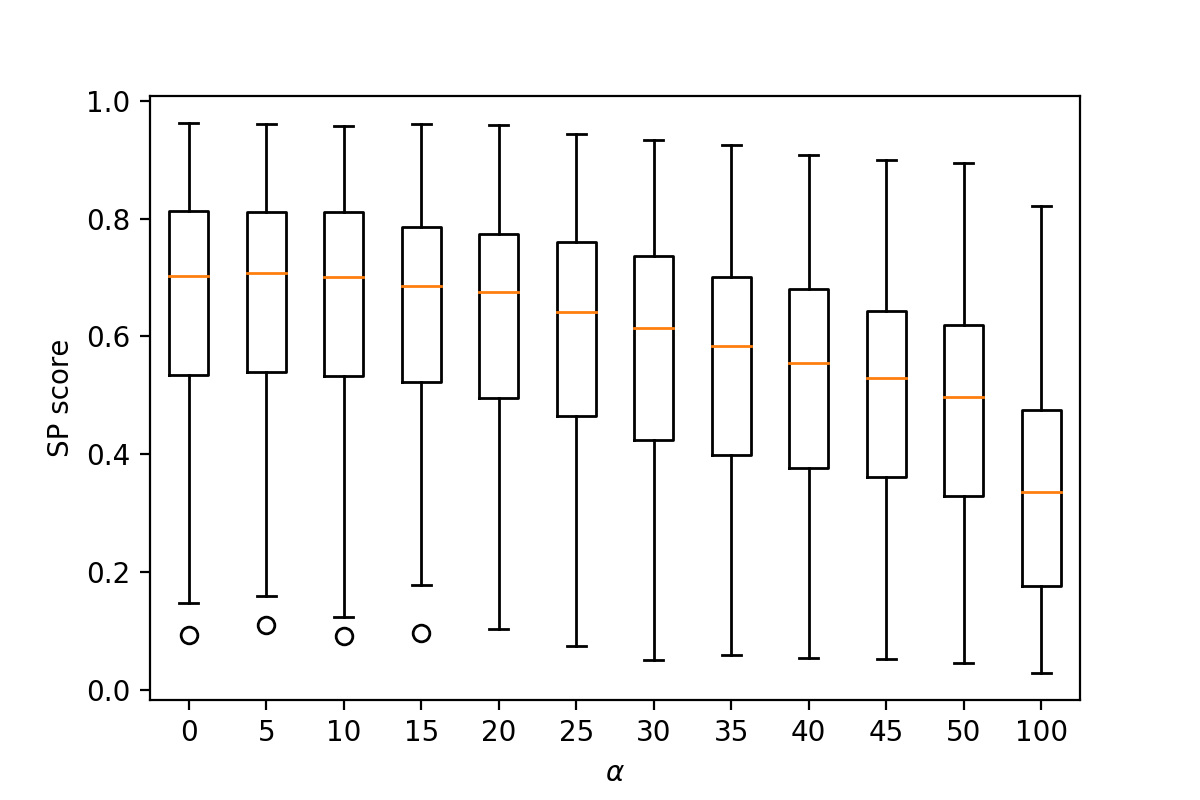

Supplement: S6 Fig — (TIF) [file pcbi.1006547.s010.tif]

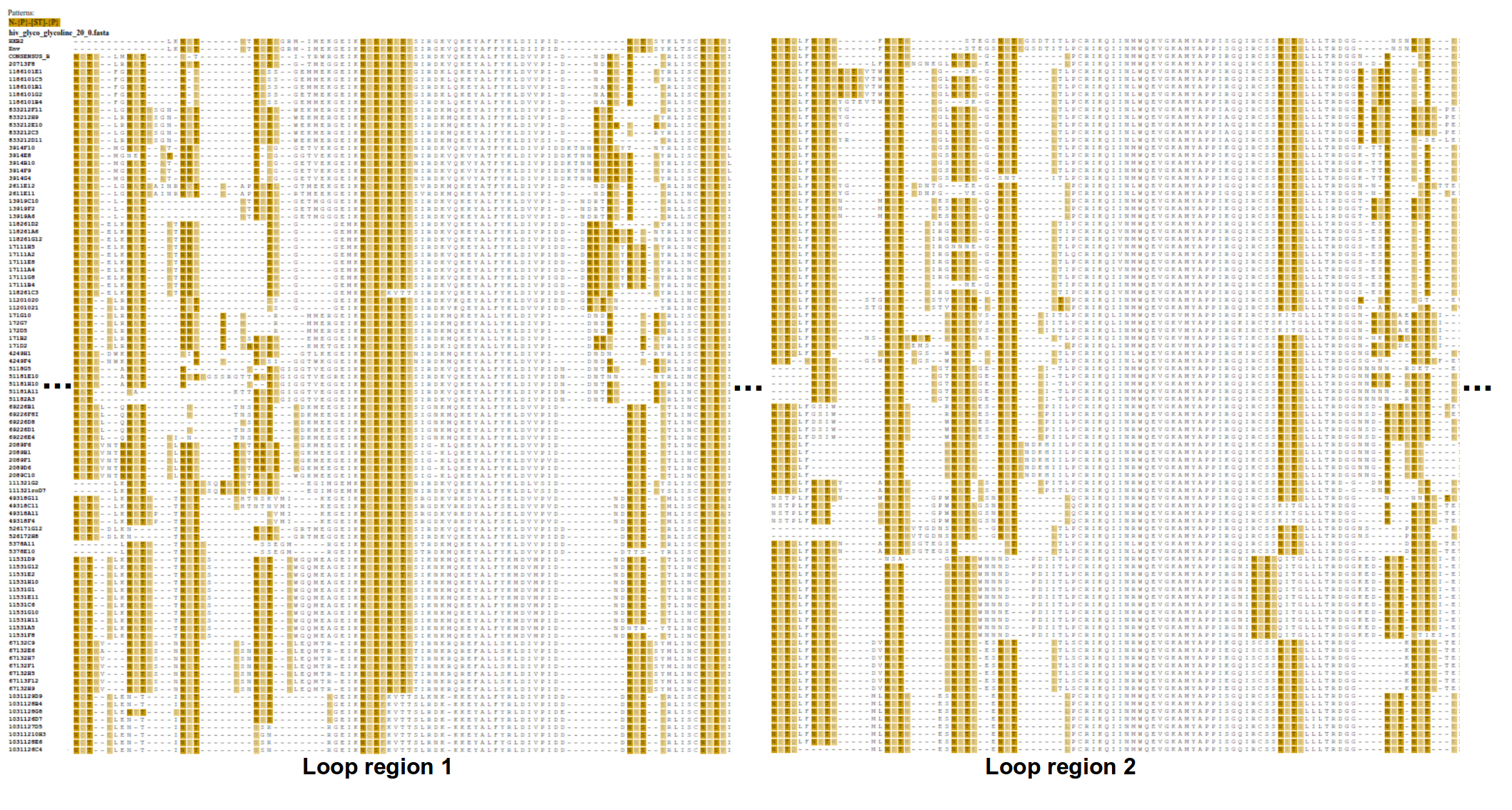

Supplement: S7 Fig — The coloured residues indicate the location of the motif, semi-transparent colours indicate the spacer residues in the motif. (TIF) [file pcbi.1006547.s011.tif]

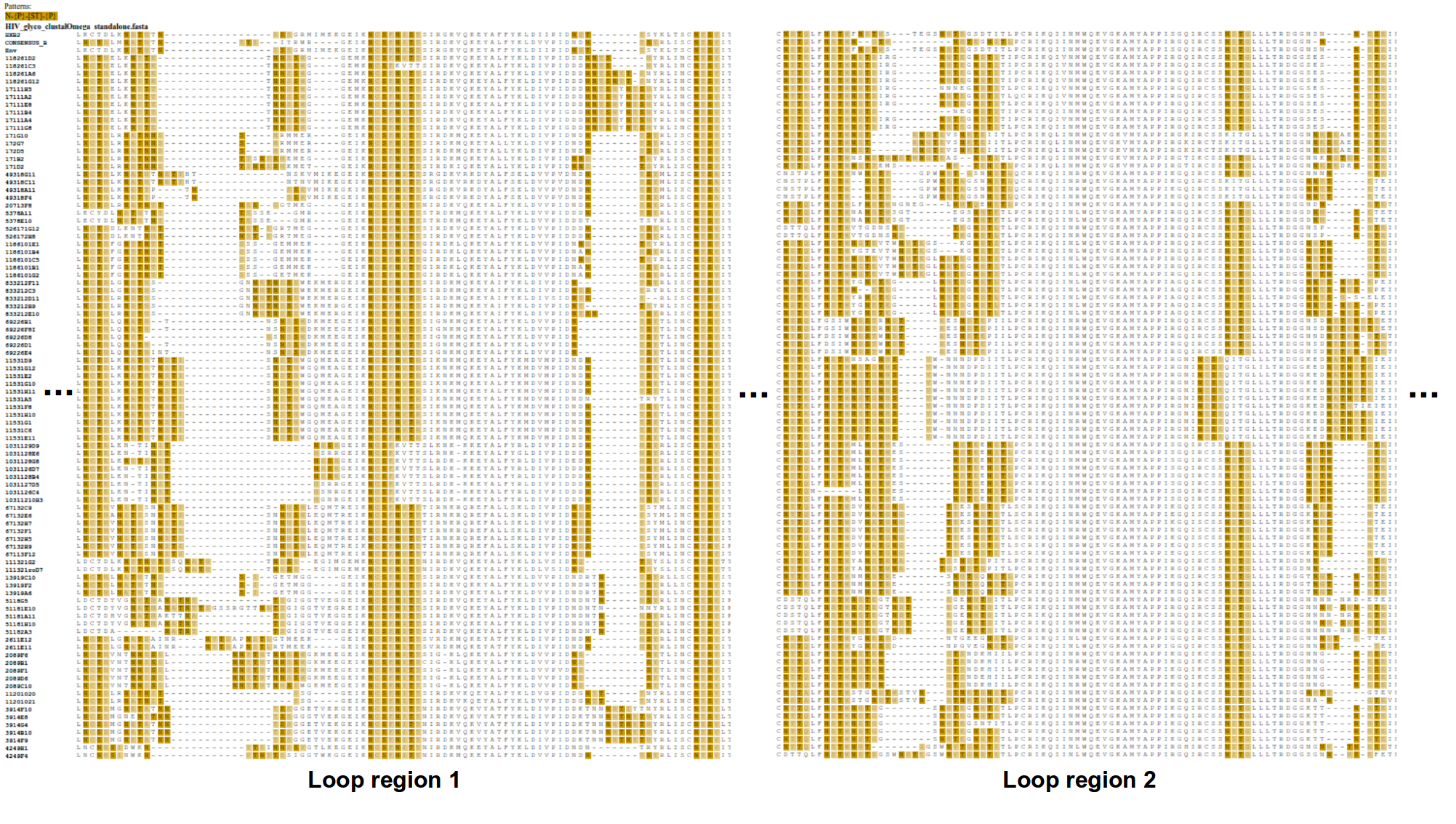

Supplement: S8 Fig — The coloured residues indicate the location of the motif, semi-transparent colours indicate the spacer residues in the motif. (TIF) [file pcbi.1006547.s012.tif]

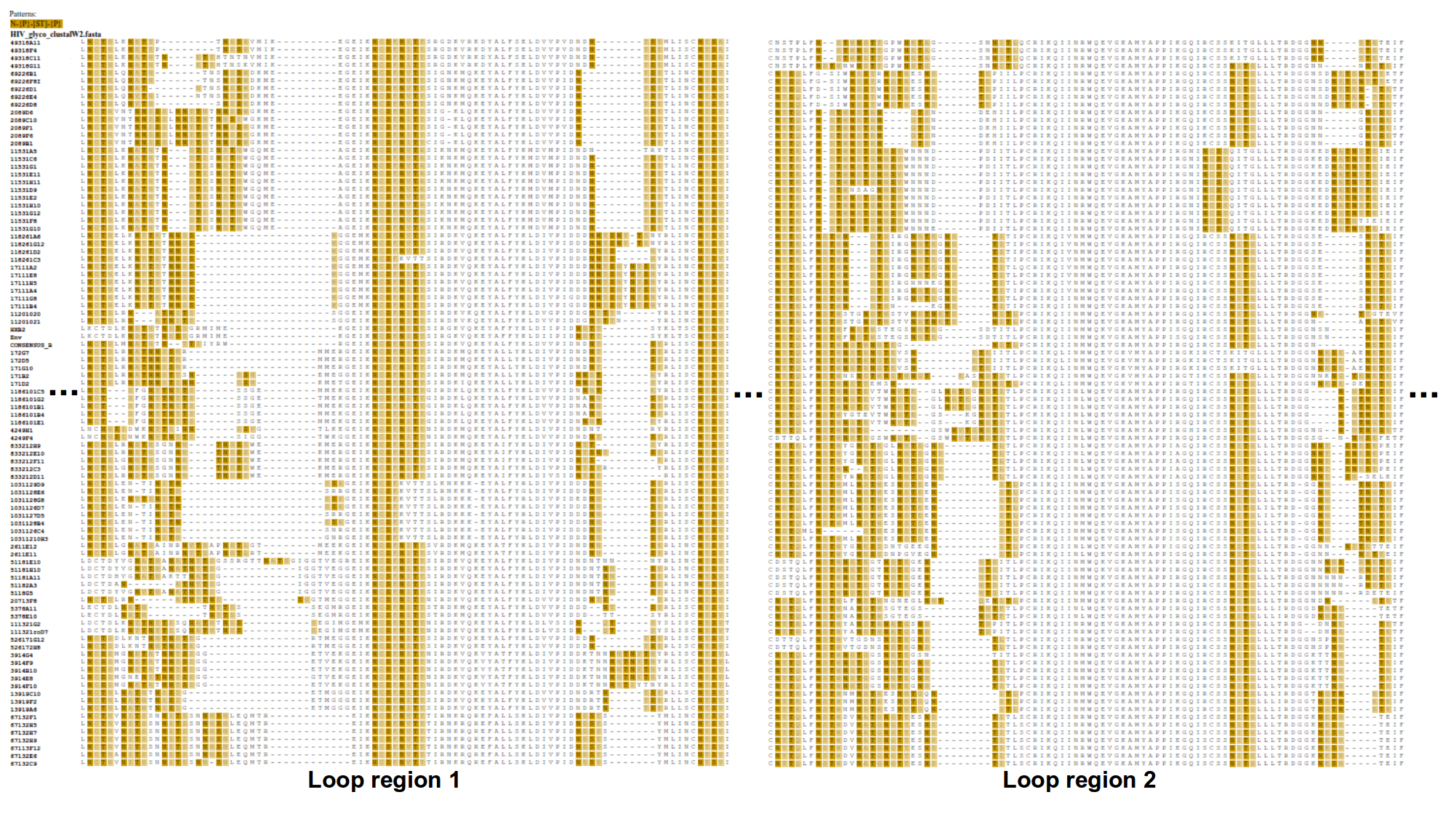

Supplement: S9 Fig — The coloured residues indicate the location of the motif, semi-transparent colours indicate the spacer residues in the motif. (TIF) [file pcbi.1006547.s013.tif]

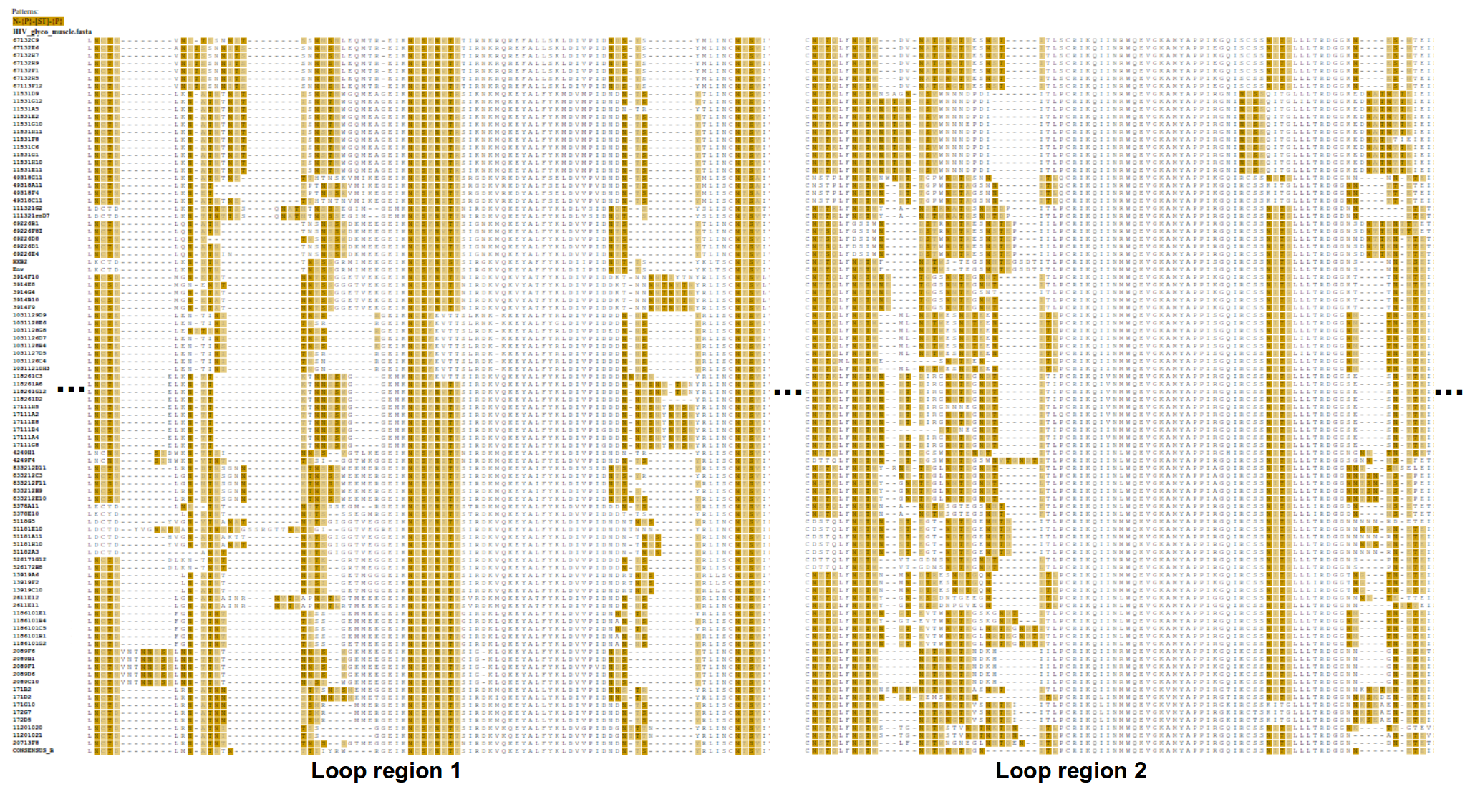

Supplement: S10 Fig — The coloured residues indicate the location of the motif, semi-transparent colours indicate the spacer residues in the motif. (TIF) [file pcbi.1006547.s014.tif]
